# Optimizing the management of thyroid specimens to efficiently generate whole slide images for diagnosis

**DOI:** 10.1007/s00428-024-03762-3

**Published:** 2024-02-14

**Authors:** Catarina Eloy, João Vale, Mariana Barros, Diana Oliveira, Morgana Mesquita, Mónica Curado, João Pinto, António Polónia

**Affiliations:** 1 Pathology Department, Medical Faculty of University of Porto, Porto, Portugal; 2 Pathology Laboratory, Institute of Molecular Pathology and Immunology of University of Porto (IPATIMUP), Rua Júlio Amaral de Carvalho 45, 4200-135 Porto, Portugal; 3 Department of Pathological, Cytological and Thanatological Anatomy, School of Health of Polytechnic Institute of Porto, Porto, Portugal; 4 i3S—Instituto de Investigação e Inovação em Saúde, Porto, Portugal; 5 Departamento de Sistemas Biofuncionais Do Corpo Humano da Escola de Medicina E Ciências Biomédicas, Inovação E Desenvolvimento, Instituto de Investigação, Fundação Fernando Pessoa (FP-I3ID), Porto, Portugal

**Keywords:** Digital pathology, Efficiency, Whole slide image, Digital workflow, Specimen inking

## Abstract

Transition from optical to digital observation requires an additional procedure in the pathology laboratory, the scanning of glass slides, leading to increased time and digital archive consumption. Thyroid surgical samples often carry the need to collect several tissue fragments that generate many slides to be scanned. This study evaluated the impact of using different inking colours for the surgical margin, section thickness, and glass slide type, in the consumption of time and archive. The series comprehended 40 nodules from 30 patients, including 34 benign nodules in follicular nodular disease, 1 NIFTP, and 5 papillary carcinomas. In 12 nodules, the dominant pattern was microfollicular/solid and in 28 it was macrofollicular. Scanning times/mm^2^ were longer in red-inked fragments in comparison to green (*p* = 0.04) and black ones (*p* = 0.024), and in blue-inked in comparison to green ones (*p* = 0.043). File sizes/mm^2^ were larger in red-inked fragments in comparison to green (*p* = 0.008) and black ones (*p* = 0.002). The dominant pattern microfollicular/solid was associated with bigger file size/mm^2^ in comparison with the macrofollicular one (*p* < 0.001). All scanner outputs increase significantly with the thickness of the section. All scanning outputs increase with the usage of adhesive glass slides in comparison to non-adhesive ones. Small interventions in thyroid sample management that can help optimizing the digital workflow include to prefer black and green inking colours for the surgical margins and 2 µm section in non-adhesive glass slides for increased efficiency.

## Introduction

The digital transformation of pathology laboratories is happening all over the world with growing introduction of the scanning process and generation of whole slide images (WSIs) for primary diagnosis [1]. The advantages of digital workflow are well-known, concerning both histological and cytological specimens, independent of their topographic origin [2–5].

The usage of WSIs for primary histological diagnosis in thyroid pathology is non-inferior to the classic usage of the optical microscope [6] provided that adequate training [7] and validation [8] procedures are undertaken.

The transition from optical to digital observation requires an additional procedure in the pathology laboratory, the scanning of glass slides. This additional procedure in tissue preparation for observation consumes more time that may contribute to an increment in turnaround time. In addition, the 2-dimensional observation of WSIs is more sensitive to poor-quality preparations than the 3-dimensional observation under the optical microscope.

Recently, our group reported that for full tissue/cell scanner detection of low contrast or paucicellular sample, such as cellblocks, there is a need to highlight the limits of the samples with ink prior to tissue processing [9]. This inking prevents samples from being incompletely represented in WSIs and subsequent false negative results. Nevertheless, this inking procedure increases scanning time and file size of the WSIs. The file size is a new laboratory parameter to be specifically controlled after the digital transformation, since costs associated with digital archive archiving can easily escalate [10]. Having this in mind, it is easy to understand that a change in the pre-scanner management of the samples needs to occur so that high-quality glass slides are produced to be efficiently scanned [11].

In this work, we test the influence of some laboratory procedures, inherent to the management of thyroid surgical specimens, in the efficiency of the high-quality scanning process. First, we evaluate the impact of using distinct colour inks during macroscopic assessment for labelling radial margin. Then, we evaluate the impact of different thicknesses of the section of the thyroid tissue embedded in paraffin block. Finally, we test the influence of the usage of two types of slides, adhesive and non-adhesive, in the scanning efficiency.

## Material and methods

### Selection of specimens and collection of tissue fragments

**Fig.1:**
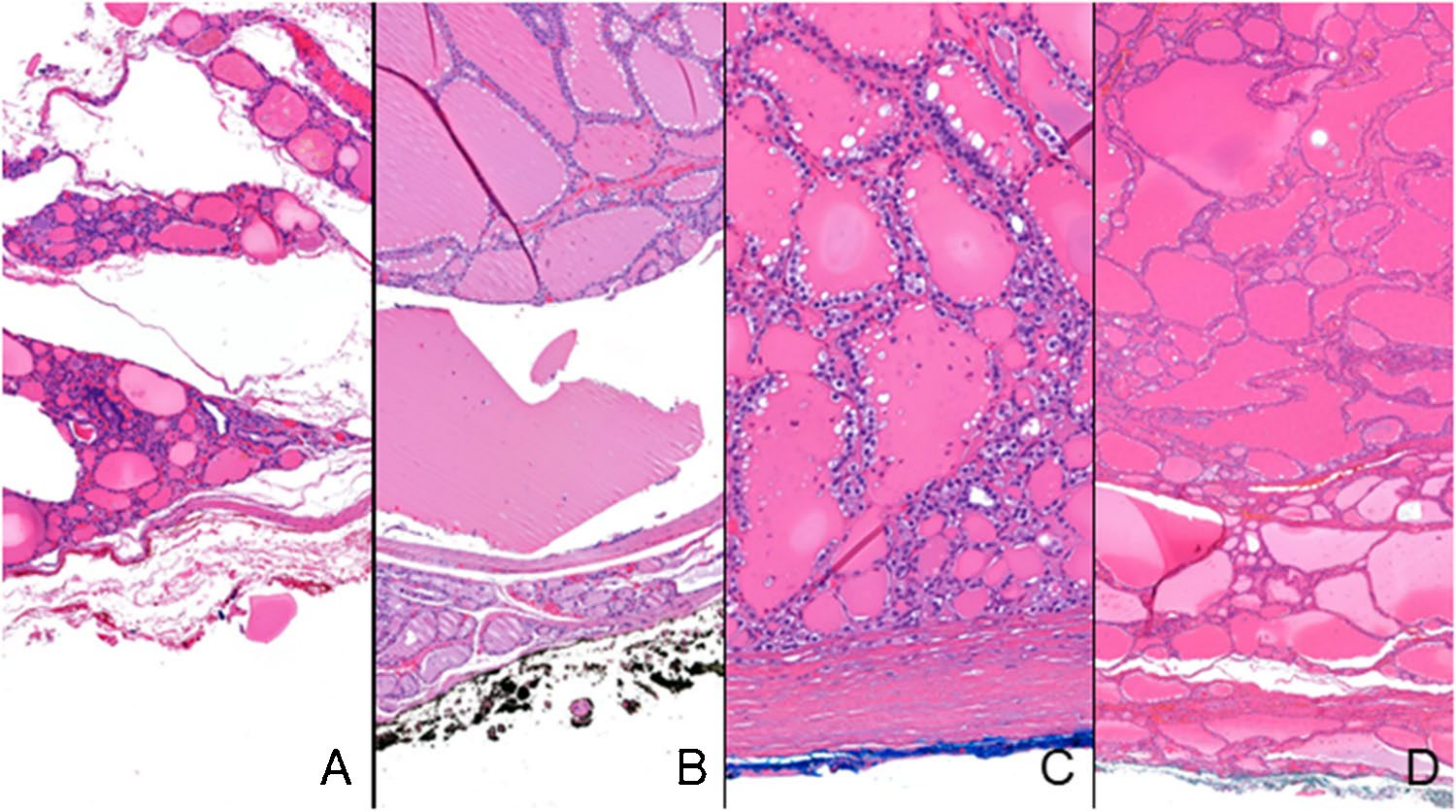
Hematoxylin and eosin (H&E) whole slide images of four cases with their surgical margins inked with different colours: **A** red, **B** black, **C** blue, **D** green

This is a prospective study that includes 30 consecutive thyroid surgical specimens received for observation at the Pathology Laboratory of Institute of Molecular Pathology and Immunology of University of Porto, from February 2023 to May 2023, that accomplished to inclusion criteria described next. In this series, the thyroid surgical specimens should contain at least one well-limited follicular derived nodule with 10 mm in largest diameter or more. This(ese) well-limited nodule(s) should be suitable to be sampled in a 10-mm(width) × 10-mm(long) × 3-mm(thickness) tissue fragment that also included the radial margin. A single fragment was collected per nodule. One to four nodules were sampled per specimen. A total of 40 fragments, corresponding to the 30 surgical specimens from 30 different patients, were collected. The radial margin represented in each fragment was selectively inked with a brush. Of the 40 fragments, 10 were inked in red, 10 were inked in black, 10 were inked in blue, and 10 were inked in green (Epredia©, Kalamazoo, USA) (Fig. 1). If two nodules from the same lobe were to be sampled and included in the study, the used ink was the same to prevent colour contamination. Afterwards, all inked surgical margins were sprayed with an inking fixative constituted by a solution of absolute ethanol with glacial acetic acid.

Each of the 40 selected tissue fragments was processed (Donatello™ series 2 tissue processor, Diapath™, Martinengo, Italy) in a single cassette, together with the remaining cassettes of the corresponding case.

### Glass slide preparation

After manual embedding, each of the 40 paraffin blocks was sectioned in a microtome HistoCore BIOCUT® (Leica Biosystems, Melbourne, UK) according to the following:

- First section: 2 µm section to be attached to a regular non-adhesive slide (Süsse Labortechnik GmbH, Gudensberg, Germany) (all nodules)
- Second section: 3 µm section to be attached to a regular non-adhesive slide (only black inked nodules)
- Third section: 4 µm section to be attached to a regular non-adhesive slide (only black inked nodules)
- Forth section: 2 µm section to be attached to an adhesive slide (TOMO slide, Matsunami Glass Ind., Ltd., Tokyo, Japan) (only black inked nodules)

All slides were stained by the hematoxylin and eosin (H&E) technique (Tissue-Tek Prisma® Plus automatic stainer, Sakura™, Tokyo, Japan) and coverslipped with film (Tissue-Tek Film®, Sakura™, Tokyo, Japan).

### Scanning, observation, and annotation procedures

All slides were scanned on the Pannoramic 1000® Scanner (3DHISTECH Ltd, Budapest, Hungary) at 20 × (0.25 µm/pixel), using the scanning protocol clinically validated for histological WSIs currently used in our laboratory and managed by SlideCenter software (3DHISTECH Ltd., Budapest, Hungary). The time for scanning and the size file for each slide were recorded.

**Fig.2:**
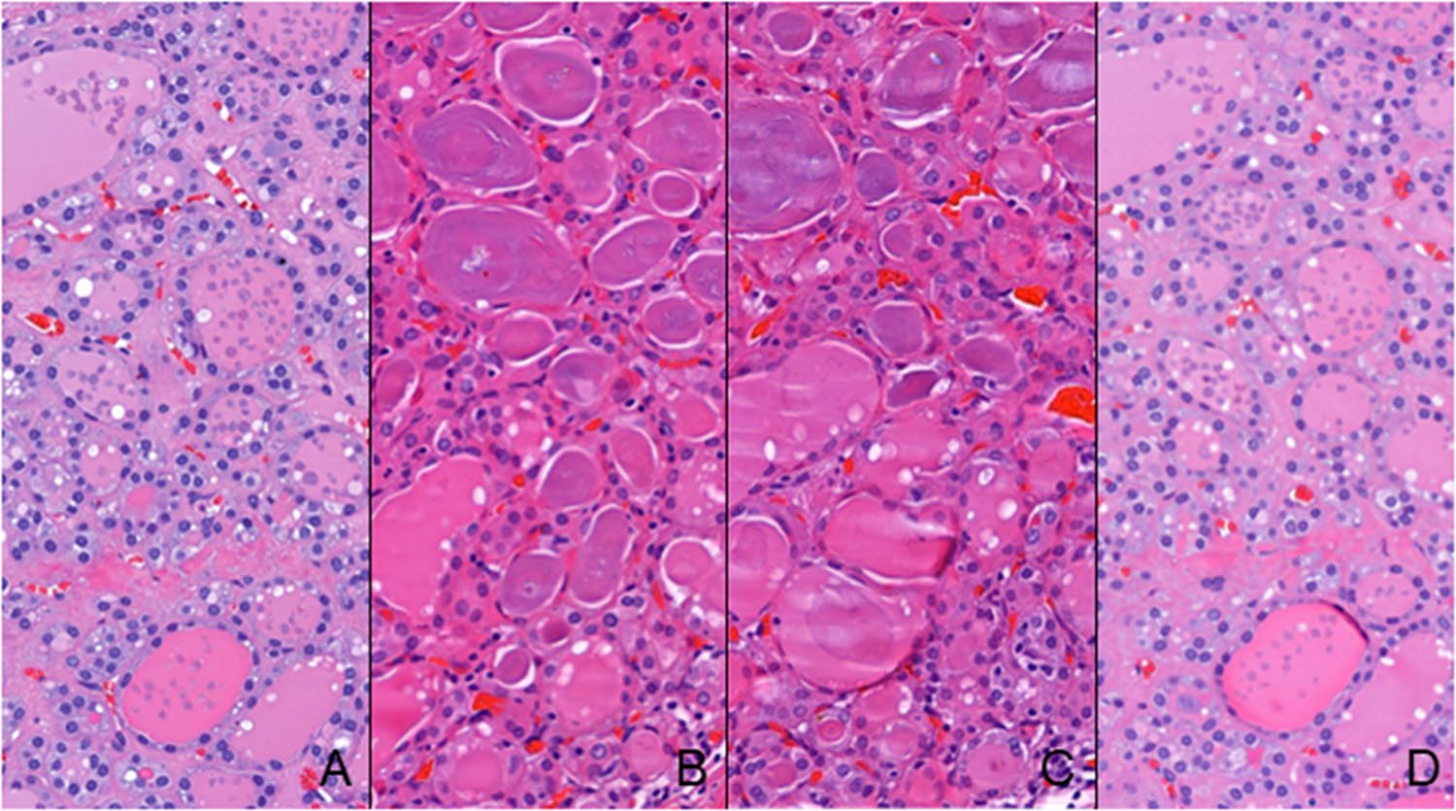
Aspect of the 4 different type of section of the same nodule disclosing the diagnosis of benign nodule in the setting of follicular nodular disease. **A** Hematoxylin and eosin (H&E), 40 × , 2 µm, non-adhesive slide; **B** H&E, 40 × , 3 µm, non-adhesive slide; **C** H&E, 40 × , 4 µm, non-adhesive slide; **D** H&E, 40 × , 2 µm, adhesive slide

**Fig. 3:**
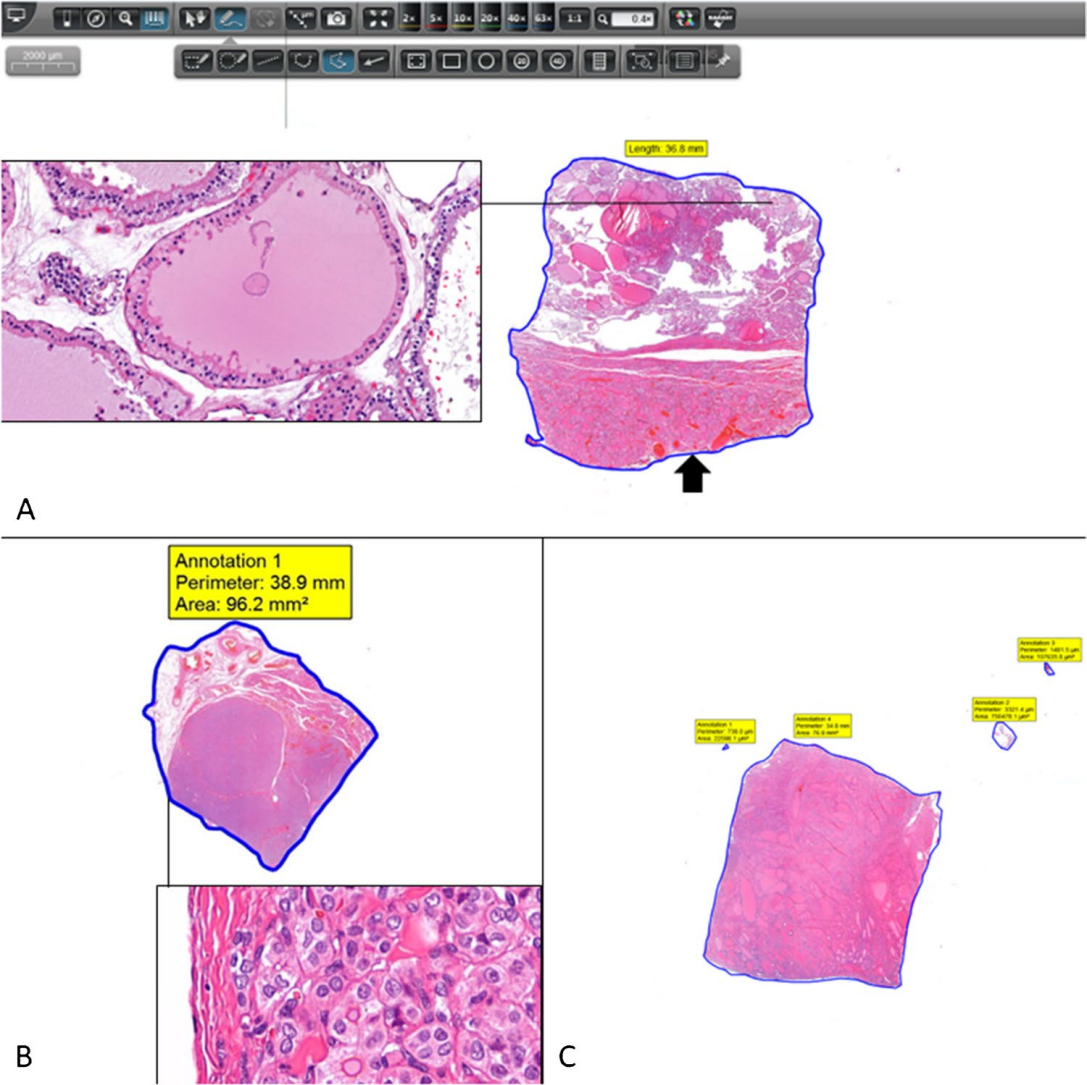
Measurement of the perimeter and area of the thyroid tissue fragments using digital tools on the whole slide image. **A** Measurement of the perimeter of a fragment inked red (arrow) that was representative of a follicular nodular disease with predominant macrofollicular growth pattern, H&E, 0.4 × , inset 41 × ; **B** measurement of the area and perimeter of a fragment inked green including a small fragment aligned with the largest fragment, this fragment was representative of a solid pattern papillary carcinoma, H&E, 0.4 × , inset 41 × ; **C** measurement of the area and perimeter of a fragment inked blue including 3 small fragments detached, this fragment was representative of a follicular nodular disease with dominant microfollicular pattern, H&E, 0.4 ×

The WSIs were included in the virtual tray of the respective case for observation by one pathologist (CE) using the SlideViewer software (3DHISTECH, Ltd., Budapest, Hungary) (Fig. 2). On top of the WSI obtained from the first Sect. (2 µm, non-adhesive slide)m a digital measurement of the tissue was obtained. At 0.4 × magnification view and using the tool “draw a closed polygon,” we calculated the area and perimeter of each fragment. Within this area were included: the larger tissue fragment and very small tissue fragments detached, that could be identified as thyroid tissue. Small tissue fragments that were aligned with the largest fragment were included in a single measurement while tissue fragments located far from the largest fragment were measured separately and summed to those of the corresponding largest fragment (Fig. 3).

All 40 nodules were observed and classified according to the 5th edition of the WHO book for Endocrine and Neuroendocrine tumours. For each nodule, the following data was recorded: age (years) and gender of the patient, largest size of the nodule (mm), location of the nodule, diagnosis, dominant histological pattern represented in the WSI, area of the fragment (mm^2^), perimeter of the fragment (mm), scanning time per each section (seconds), file size of each WSI per section (Mb), scanning time per area per section (seconds/mm^2^), and file size per area per section (Mb/mm^2^).

### Statistical analysis

Statistical analyses were performed using the Statistical Package for the Social Sciences (SPSS) version 29.0 for Windows. Fisher’s exact test was used for comparison of qualitative variables. The pair *t* test, the one-way ANOVA, and the Kruskal–Wallis test (KW) were used for comparison of quantitative variables, after testing for normal distribution through the Kolmogorov–Smirnov test. The level of significance was set at *p* < 0.05.

## Results

The prospective series studied in this work concerns 40 nodules from 30 patients described in detail in Table 1. The patients were 24 females and 6 males with ages between 27 and 75 years old, median 60.5 years [percentile 25: 45.5 years, percentile 75: 65.0 years].

**Table 1:** Features of the nodules included in each inking colour group

Features	Inking colour of the surgical margin				*p*
	Red	Black	Blue	Green	
Patient					
Gender	9 F:1 M	8 F:2 M	9 F:1 M	6 F:4 M	n.a
Age (years) ^a^	46.0 [37.0–58.3]	63.0 [61.0–65.3]	62.5 [44.8–71.0]	60.5 [52.3–64.3]	n.a
Nodule, *n* (%)	10 (25)	10 (25)	10 (25)	10 (25)	
Size (mm) ^a^	12.0 [10.8–33.3]	22.5 [2.05–47.5]	31.0 [16.5–61.5]	20.5 [15.8–36.3]	n.a
Location					n.a
Right lobe	1	2	6	9	
Isthmus	1	0	1	0	
Left lobe	8	8	3	1	
Dominant pattern					**0.007**^b^/0.141^c^
Macrofollicular	4	9	6	9	
Microfollicular/solid	6	1	4	1	
Diagnosis					n.a
FND	7	10	8	9	
NIFTP	1	0	0	0	
Papillary carcinoma	2	0	2	1	
Fragment measurements^a^					
Perimeter (mm)^d^	39.4 ± 4.0	40.5 ± 3.9	38.6 ± 4.1	38.9 ± 3.0	0.695^e^
Area (mm^2^) ^d^	97.5 ± 21.2	90.4 ± 16.9	87.5 ± 13.2	91.6 ± 13.8	0.597 ^e^
Scanning outputs ^f^					
Time (seconds) ^a^	56.5 [54.0–75.8]	44.5 [39.0–48.0]	53.0 [44.8–58.3]	44.5 [37.8–49.8]	**0.002** g
Time/perimeter (seconds/mm)^a^	1.48 [1.29–1.83]	1.11 [0.97–1.17]	1.36 [1.12–1.65]	1.09 [0.99–1.24]	** < 0.001**^ h^
Time/area (seconds/mm^2^)^a^	0.64 [0.58–0.69]	0.50 [0.47–0.52]	0.57 [0.50–0.75]	0.47 [0.44–0.53]	** < 0.001**^ h^
File size (Mb) ^d^	729.7 ± 179.3	495.1 ± 8.03	590.8 ± 87.8	541.4 ± 158.6	**0.003** ^i^
File size/perimeter (Mb/mm) ^d^	18.53 ± 4.00	12.19 ± 1.39	15.50 ± 3.04	13.75 ± 3.20	** < 0.001** ^j^
File size/area (Mb/mm^2^) ^d^	7.60 ± 1.59	5.53 ± 0.47	6.85 ± 1.22	5.83 ± 0.99	** < 0.001** ^j^

Legend: *F* female, *M* male, *FND* benign nodule in the setting of follicular nodular disease, *n.a* not available due to the small number of cases; ^a^expressed as median [percentile 25-percentile 75], ^b^comparison between the dominant pattern of the red-inked fragments and the one of the group black plus green inked nodules (Fisher’s exact test),^c^comparison between the dominant pattern of the blue-inked fragments and the one of the group black plus green inked nodules (Fisher’s exact test), ^d^expressed as mean ± standard deviation, ^e^one way ANOVA test, ^f^scanning outputs were measured during the scanning of 2 µm non-adhesive slides, ^g^the Kruskal–Wallis test [Dunn test with Bonferroni correction demonstrated that mean scanning times are similar in paired comparisons except for the red coloured fragments that take more time to scan in comparison with the green (*p* = 0.01) and with the black coloured ones (*p* = 0.011)], ^h^the Kruskal–Wallis test [Dunn test with Bonferroni correction], ^i^one-way ANOVA test [demonstrated that average size file is similar in paired comparisons except for the red coloured fragments that are larger in comparison with the green (*p* = 0.02) and with the black coloured ones (*p* = 0.002)], ^j^one-way ANOVA test

The bold significance *p*<0.05

The 40 nodules had a median size of 22.5 mm [percentile 25:15.0 mm, percentile 75: 39.3 mm] and ranged from 10 to 75 mm in largest dimension. The nodules were located in the right lobe in 18 cases, in the isthmus in 2 cases and in the left lobe in 20 cases.

In 12 nodules, the dominant pattern represented in the fragment was microfollicular/solid while the remaining 28 nodules disclosed a predominant macrofollicular pattern. The diagnoses of the nodules were distributed as follows: 34 benign nodules in the setting of follicular nodular disease, 1 non-invasive follicular thyroid neoplasm with papillary-like nuclei (NIFTP), and 5 solid subtypes of papillary carcinoma.

The perimeters of the tissue fragments ranged from 31.3 to 48.2 mm, measured on average 39.4 ± 3.7 mm and were similar among the differently inked fragments (*p* = 0.695). The areas of the tissue fragments ranged from 56.8 to 144.6 mm^2^, measured in average 91.7 ± 16.4 mm and are similar among the differently inked fragments (*p* = 0.597).

Scanning parameters were recorded during the capture of the WSIs obtained after the 2 µm section in a non-adhesive slide. Scanning times ranged from 33 to 163 s and lasted a mean time of 49.5 s [percentile 25: 41.5 s, percentile 75: 56.8 s]. Scanning times were similar among the differently inked fragments except for the red ones that took longer times to scan in comparison to the green (*p* = 0.01) and the black ones (*p* = 0.011).

Scanning times/mm (perimeter units) ranged from 0.96 to 4.12 s/mm and lasted a median time of 1.21 s/mm [percentile 25: 1.05 s/mm, percentile 75: 1.45 s/mm]. Scanning times/mm were similar among the differently inked fragments except for the red ones that took longer time to scan/mm (perimeter units) in comparison with the green (*p* = 0.006) and the black (*p* = 0.001).

The file size/mm (perimeter units) of the WSIs ranged from 9.63 to 25.78 Mb/mm and measured a mean size of 14.99 ± 3.78 Mb/mm. File sizes/mm were similar among the differently inked fragments except for the red ones that were larger than the green (*p* = 0.008) and the black (*p* < 0.001).

Scanning times/mm^2^ ranged from 0.41 to 1.59 s and lasted a mean time of 0.53 s [percentile 25: 0.48 s, percentile 75: 0.63 s]. Scanning times/mm^2^ were similar among the differently inked fragments except for the red ones that took longer times to scan/mm^2^ in comparison to the green (*p* = 0.04) and the black ones (*p* = 0.024), as well as the blue that took longer times to scan/mm^2^ than the green ones (*p* = 0.043). The file size of the WSIs ranged from 339 to 1021 Mb and measured on average 589.3 ± 156.2 Mb. File sizes were similar among the differently inked fragments except for the red that were larger in comparison to the green (*p* = 0.02) and the black ones (*p* = 0.002). The file size/mm^2^ of the WSIs ranged from 4.54 to 9.97 Mb and measured on average 6.45 ± 1.38 Mb. File sizes/mm^2^ were similar among the differently inked fragments except for the red that were larger in comparison to the green (*p* = 0.008) and the black ones (*p* = 0.002).

Since both microfollicular/solid dominant pattern and scanner outputs were increased in the red-inked group of fragments, a multivariate analysis (multivariate linear regression) was performed to understand the interference of these variables (Table 2). In the overall, the inking colour of the fragments appears to be the determinant factor on the values obtained in all the scanning outputs; nevertheless, the dominant pattern appears to be also determinant in the file size/mm^2^ being the microfollicular/solid patten associated with a larger file size per area in comparison with the macrofollicular one [8.02 ± 1.23 Mb/mm^2^ versus 5.77 ± 0.74 Mb/mm^2^, *p* < 0.001(*T* test), respectively].

**Table 2:** Results of the multivariate linear analysis separating the influence of the inking colour and the dominant pattern on the scanning outputs

Scanning outputs	Adjusted coefficient			
	*β*	CI (95%)	*R*^2^	*p*
Time (seconds)			0.166	
Dominant histological pattern	0.059	[–12.661; 18.174]		0.719
Inking colour of the surgical margin	0.428	[1.874; 14.513]		**0.012**
Time/area (seconds/mm^2^)			0.192	
Dominant histological pattern	0.207	[–0.051; 0.233]		0.203
Inking colour of the surgical margin	0.355	[0.005; 0.122]		**0.033**
File size (Mb)			0.320	
Dominant histological pattern	0.265	[–11.002; 189.503]		0.079
Inking colour of the surgical margin	0.429	[18.164; 100,346]		**0.006**
File size/area (Mb/mm2)			0.641	
Dominant histological pattern	0.616	[1.185; 2.469]		** < 0.001**
Inking colour of the surgical margin	0.324	[0.131; 0.657]		**0.004**

Multivariate linear regression analysis: *CI* confidence interval

The bold significance *p*<0.05

**Fig. 4:**
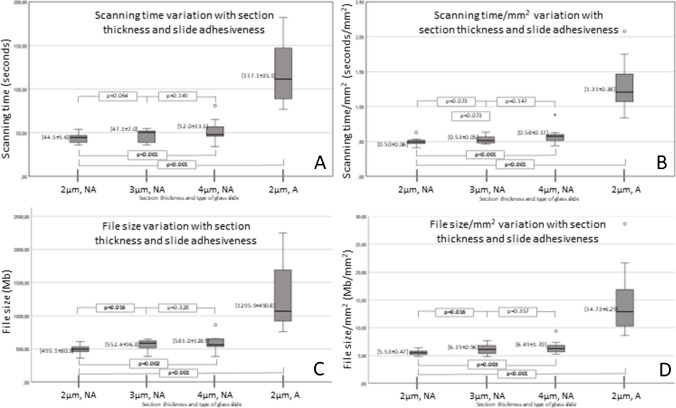
Graphic illustration of the variation of the scanning time and file size of whole slide images per section thickness, type of slide, and according to the tissue area. Graphic **A** illustration of the mean values ± standard deviation of scanning time variation with the section thickness and type of glass slide. Graphic **B** illustration of the mean values ± standard deviation of scanning time per area of the fragment according to the section thickness and type of glass slide. Graphic **C** illustration of the mean values ± standard deviation of file size variation with the section thickness and type of glass slide. Graphic **D** illustration of the mean values ± standard deviation of file size per area of the fragment according to the section thickness and type of glass slide. A adhesive glass slide, NA non-adhesive glass slide. The comparison of the results was performed using the pair *T* test

Concerning the variation of the scanning outputs with the thickness of the section, it is possible to understand that all outputs progressively increase with the increasing thickness of the section, being these differences significative, namely when comparing the 2 µm section with the 4 µm section (scanning time *p* < 0.001, scanning time/mm^2^ *p* < 0.001, file size *p* = 0.002, and file size/mm^2^ *p* = 0.003) (Fig. 4).

The usage of an adhesive glass slide also leads to a significant increment in all scanning outputs in comparison to the usage of a non-adhesive glass slide, for the same 2 µm thickness of section (scanning time *p* < 0.001, scanning time/mm^2^ *p* < 0.001, file size *p* < 0.001, and file size/mm^2^ *p* = 0.001) (Fig. 4).

## Discussion

Together with the process of digital transformation, there is the need for controlling specific parameters of the laboratory functioning that are related with quality, turnaround time, and costs, namely digital archive-related costs [1, 2, 10]. As previously demonstrated by our group, delicate interventions in the sample management, such as inking samples [9] or automize coverslipping [12], may dramatically interfere with these parameters.

Taking into consideration that surgical samples often require the collection of many fragments, their analysis may be associated to high consumption of resources. Thyroid surgical samples are frequently studied in pathology laboratories, and, by virtue of their complex diagnosis, may require total embedding of the periphery of well-limited nodules [13] carrying the need to collect a large amount of fragments and respective WSIs. In this study, we evaluated, specifically in the setting of thyroid surgical samples, the impact of the usage of different inking colours for the surgical margin, section thickness, and glass slide type, in the consumption of time and digital archive.

Inking the surgical margin is a regular procedure undertaken at the macroscopic observation for microscopic labelling of the limits of the specimens and consequent evaluation of the extent of the surgical procedure. As demonstrated previously, inked specimens take longer to scan and consume more digital archive than those not inked [9]. Since inking provides relevant information to the patient management, these authors are not suggesting stopping inking thyroid surgical specimens but, instead, to search for the inking colour that takes less time to scan and carries smaller file size. To control the influence of the size of the tissue fragment in the scanning time and file size, a precise measure of the perimeter and area of each fragment was obtained to calculate the results per mm^2^. In addition, records of the dominant pattern of the nodule were also obtained to prevent bias of the results associated with the nodule cellularity. Cellularity of microfollicular/solid lesions contributes to an increment of the details of the image and may eventually lead to generate more information at the pixel level in comparison with less cellular macrofollicular/colloid-rich lesions. In fact, in this study, the microfolicular/solid pattern was significantly associated with a larger file size/mm^2^. This finding is not indicative that less sampling should be undertaken during the macroscopic observation of microfollicular/solid patterned nodules. Instead, this finding serves as a reference to further studies to undergo cellularity control while studying scanning outputs. In this study, as well as before [9], the usage of black and green colours appears to result in similar scanning time and file size. On the other hand, the red and blue inking results in larger scanning time/mm^2^ while red inking also results in larger file size/mm^2^. The same holds true for scanning time/mm (perimeter units).

As stated above, cellularity of the tissues may influence the scanning time and the file size of WSIs. Since the section thickness tends to represent more cells at different planes as the thickness increases, a paired evaluation of the scanning time and file size of the same nodules consecutively section at 2 µm, 3 µm, and 4 µm (assumed as the reasonable range of section thickness used for routine in pathology laboratories) was undertaken. In this work, we demonstrate that scanning time and file size tend to increase significantly with the section thickness, namely when comparing 2 µm with 4 µm. To warrant a flatter (2-dimensional) WSI with less time and archive consumption, we flag the 2 µm section as the best choice. A 2 µm section exercise may be challenging to the less experienced microtomist but is feasible after training, according to the experience of the authors. For similar reasons, the usage of a non-adhesive glass slide consumes less scanning time and less file size in comparison with the usage of adhesive glass slides that retain all sorts of small particles incrementing the capture extent. This is probably one of the reasons that justifies the large size of the immunohistochemical files in comparison with respective H&E, since immunohistochemistry is usually performed in adhesive glass slides (non-reported observations).

We recognize as limitations of this study the small size of the sample and the usage of a single type of scanner. Larger series studies in multiple scanners could strengthen our observations.

To properly understand the causes underlying the influence of colour in scanning time and file size, it requires a study comparing different brands of ink with distinct physical/particles features. This is a limitation of the current study that may be explored in future research on the issue, allowing a characterization of the materials that better contribute to generate adequate glass slides and respective whole slide images. We speculate that the inherent features of the ink, including the capacity to spread along the tissue, as well as the respective pixel-related information, may contribute to these results.

As a summary, we emphasize that small interventions in the tissue sample management before scanning can optimize the digital workflow, reducing time and costs. These interventions, specifically in thyroid specimens, may include the preference in using black and green inking colours for the surgical margins and the usage of 2 µm section in non-adhesive glass slides for increased efficiency.

## Data Availability

Data generated or analyzed during this study are partially included in this published article and may be provided after reasonable request.
